# Prediabetes in Transition: Insights from the Tehran Lipid and Glucose Study

**DOI:** 10.5812/ijem-166455

**Published:** 2025-10-31

**Authors:** Zahra Bahadoran, Farhad Hosseinpanah

**Affiliations:** 1 Micronutrient Research Center, Research Institute for Endocrine Disorders, Research Institute for Endocrine Sciences, Shahid Beheshti University of Medical Sciences, Tehran, Iran; 2 Obesity Research Center, Research Institute for Endocrine Sciences, Shahid Beheshti University of Medical Sciences, Tehran, Iran

**Keywords:** Prediabetes, Glycemic Phenotypes, Progression, Regression, Lifestyle Interventions

## Abstract

**Context:**

Prediabetes (Pre-DM) is an intermediate state between normoglycemia and type 2 diabetes (T2D), encompassing heterogeneous phenotypes, including isolated impaired fasting glucose (iIFG), isolated impaired glucose tolerance (iIGT), and combined iIFG-impaired glucose tolerance (IFG-IGT). This review aimed to synthesize evidence from the Tehran Lipid and Glucose Study (TLGS) on modifiable and non-modifiable determinants of Pre-DM transition.

**Evidence Acquisition:**

Relevant TLGS publications were identified through PubMed and Scopus up to September 2025 using keywords including “prediabetes,” “impaired fasting glucose,” “impaired glucose tolerance,” “diabetes,” “regression,” “progression,” “determinants,” and “Tehran Lipid and Glucose Study.” Eligible studies included prospective analyses of Pre-DM regression and progression.

**Results:**

Evidence from the Tehran Lipid and Glucose Study indicates that both non-modifiable and modifiable factors influence the Pre-DM transition. Regression to normoglycemia (NGR) was associated with younger age, lower fasting glucose, higher physical activity, and adherence to healthy diets, whereas progression to T2D was associated with obesity, central adiposity, the combined IFG-IGT phenotype, and unhealthy dietary patterns. Specifically, high adherence to the Alternate Healthy Eating Index (AHEI), higher intakes of low-fat dairy, certain minerals, and coffee facilitate regression to NGR, while Western-style diets increase the risk of progression to T2D. Across the phenotypes, regression to NGR was most likely in individuals with IGT, particularly those with ≥ 5% weight loss and adherence to healthy dietary patterns. Combined IFG-IGT benefited from high adherence to the AHEI. The iIFG phenotype appears particularly susceptible to excessive exposure to nitrate/nitrite, zinc, and diets high in cholesterol, saturated, and trans fats, which may attenuate reversion to NGR and facilitate progression to T2D.

**Conclusions:**

Findings from the TLGS cohort show that healthy lifestyle factors favor regression to NGR, whereas obesity and poor dietary habits accelerate progression to T2D.

## 1. Context

Prediabetes (Pre-DM) is an intermediate state between normoglycemia and type 2 diabetes (T2D), encompassing heterogeneous phenotypes, including isolated impaired fasting glucose (iIFG), isolated impaired glucose tolerance (iIGT), and combined iIFG-impaired glucose tolerance (IFG-IGT) (1, 2). Globally, Pre-DM is increasing rapidly (3, 4), and it is estimated to reach 587 million individuals by 2045 (5), with an annual rate of 5 - 10% progressing to T2D (6). In 2021, the worldwide prevalence of iIFG and iIGT was approximately 5.8% (298 million people) and 9.1% (469 million people), respectively, and these rates are expected to increase to 6.5% and 10% by 2045 (7). Prediabetes is highly prevalent and continues to rise among Iranian adults, underscoring a pressing public health challenge in the region (4, 8). A national study using 2016 and 2021 STEPS data demonstrated that the prevalence of Pre-DM increased from 23.9% to 29.0% in men and from 22.9% to 30.9% in women (9). The overall incidence of Pre-DM was reported as 46.1 and 36.8 per 1,000 person-years in men and women, respectively, with iIFG being the most common phenotype (4). Excess body weight, particularly central obesity, older age, and family history of diabetes are among the strongest predictors of Pre-DM (10). An unhealthy lifestyle, including low physical activity, unhealthy diet, and smoking, along with comorbid conditions such as hypertension and dyslipidemia, further elevate the risk (4, 8, 11). Among women, a history of maternal early menopause, history of pregnancy loss, and being divorced or widowed have been reported as additional predictors of Pre-DM (4, 12, 13). A higher educational level and being single were protective against Pre-DM incidence in men (4). These findings point to the multifaceted nature of Pre-DM, where biological, lifestyle, and social factors converge to influence its development and progression. The Tehran Lipid and Glucose Study (TLGS) is a large-scale, long-term prospective cohort in the Middle East designed to investigate risk factors for non-communicable diseases in the Iranian population (14). The TLGS is among the first cohort studies worldwide to provide pioneering evidence on modifiable and non-modifiable determinants of Pre-DM regression and progression, specifically across distinct glycemic phenotypes. This narrative review was conducted by synthesizing published findings from the TLGS that investigated determinants of Pre-DM progression to T2D and regression to normoglycemia (NGR).

## 2. Evidence Acquisition

Relevant articles were identified through a comprehensive search of TLGS publications using PubMed and Scopus up to September 2025, with no language restrictions. The search keywords included “prediabetes,” “impaired fasting glucose,” “impaired glucose tolerance,” “diabetes,” “regression,” “progression,” “risk factors,” “determinants,” and “Tehran Lipid and Glucose Study.” Eligible studies included prospective analyses examining non-modifiable (i.e., age, sex, and Pre-DM phenotype) and modifiable (i.e., adiposity measures, physical activity level, and diet) determinants of Pre-DM transition. The search identified 16 studies, most of which focused on dietary factors, and their findings were narratively summarized to provide an integrated perspective on the heterogeneity of Pre-DM trajectories.

## 3. Results

### 3.1. Prediabetes Definition and Its Phenotypes

Prediabetes is defined by elevated fasting plasma glucose (FSG, 100 - 125 mg/dL), 2-hour plasma glucose (2h-SG, 140-199 mg/dL), or glycated hemoglobin (HbA1c, 5.7 - 6.4%) (1). This definition is based on the American Diabetes Association (ADA) criteria, which lowered the IFG cutoff to 100 - 125 mg/dL (compared with the World Health Organization range of 110 - 125 mg/dL) in 2003, to reduce the discrepancy in the predictive value of IFG and impaired glucose tolerance (IGT) for the incidence of T2D (15). The ADA Expert Committee reported that this revision increased the accuracy of predicting future T2D risk. In addition, the ADA thresholds provide greater cross-population comparability and more efficiently identify individuals at elevated risk of progression to T2D (15). Isolated iIFG is defined by a fasting serum glucose (FSG) level of 100 - 125 mg/dL while maintaining normal glucose tolerance (NGT), indicated by a 2-hour serum glucose (2h-SG) below 140 mg/dL. Individuals with iIFG typically show elevated FSG and a rapid rise in serum glucose within the first hour of an oral glucose tolerance test (OGTT), which returns to normal by the two-hour mark (16, 17). This phenotype is primarily driven by impaired early-phase insulin secretion and moderate hepatic insulin resistance, with preserved skeletal muscle sensitivity (17, 18). Individuals with IFG had an estimated 4.6-fold higher pooled relative risk [95% confidence interval (CI) = 2.47 - 6.85] of progressing to T2D compared with those who had normal glucose levels (19). In the TLGS cohort, β-cell functional impairment, assessed via the homeostasis model assessment (HOMA-B < 25th percentile), was associated with the development of iIFG exclusively in men (20). Isolated impaired glucose tolerance presents with normal fasting glucose (NFG, i.e., FSG < 100 mg/dL) but elevated serum glucose throughout the OGTT, staying within the 140-199 mg/dL range even at 120 minutes (17, 21). This prolonged hyperglycemia is largely due to defects in late-phase glucose-stimulated insulin secretion (GSIS) and insulin resistance in skeletal muscle (22). Impaired gluconeogenesis does not appear to play a central role in iIGT pathogenesis; instead, reduced insulin-mediated glucose uptake, which worsens β-cell dysfunction, is thought to be the key underlying mechanism (23). The pooled relative risk of developing T2D was 5.52 (95% CI = 3.13 - 7.91) for individuals with iIGT compared with those with normoglycemia, exceeding the risk observed in subjects with iIFG (19). Data from the TLGS provide further insight, linking the extent of return to fasting state after an OGTT to future risk of Pre-DM and T2D (24). In a cohort of 4,971 normoglycemic adults followed for a median of 11.5 years, even modest elevations of 2h-SG above FSG (≥ 9 mg/dL) were associated with significantly increased risk of incident Pre-DM and T2D, with hazard ratios (HRs) of 1.26 for 9 - 16 mg/dL, 1.32 for 17-24 mg/dL, and 1.69 for ≥ 25 mg/dL differences (24). The effect was more pronounced in women and remained significant after adjustment for insulin resistance (HOMA-IR), even among individuals with low-normal FSG (< 90 mg/dL) (24). Collectively, these findings highlight that delayed return of post-load glucose toward fasting levels during an OGTT, as observed in isolated IGT, serves as an early marker of dysglycemia and a strong predictor of progression to T2D (24). In a 10-year follow-up of 1,329 participants with Pre-DM in the TLGS, individuals with iIGT were more likely to regress to NGR compared to those with iIFG (HR = 1.26, 95% CI = 1.05 - 1.51), whereas no difference was observed in the rate of progression to T2D (25).

Combined IFG-IGT occurs when both fasting glucose (100 - 125 mg/dL) and 2-hour glucose tolerance (140 - 199 mg/dL) are abnormal in the same person. The combined IFG-IGT phenotype exhibits both hepatic and peripheral insulin resistance and progressive β-cell dysfunction (2, 18). Notably, approximately 30 - 45% of individuals with IFG also present IGT, and approximately 20 - 25% of those with IGT display IFG (7), underscoring the overlap across the Pre-DM spectrum. Elevated fasting insulin levels and high insulin resistance, as measured by the HOMA-IR (> 75th percentile), have been identified as strong predictors of the combined IFG-IGT phenotype in the TLGS cohort (20). Compared with normoglycemic individuals, the pooled relative risk of developing T2D was estimated to be 12.1 (95% CI = 4.3 - 20.0) for combined IFG-IGT (19). Individuals with combined IFG-IGT had a significantly higher risk of progressing to T2D compared with those with iIFG, with a relative risk ratio (RRR) of 2.54 (95% CI = 1.71 - 3.77) in the TLGS cohort (26).

### 3.2. Predictors of Prediabetes Progression and Regression

Over 1 to 5 years, regression from Pre-DM to NGR has been reported in 33 - 59% of individuals across 47 studies (18). The lifetime risk of progression from Pre-DM to T2D has been estimated to be 57.5% and 46.1% in women and men, respectively (27). In the TLGS cohort, a 10-year follow-up of adults with Pre-DM showed that approximately 40% of participants regressed to NGR, while a comparable proportion progressed to T2D (26). Various factors may affect the likelihood of Pre-DM regressing to NGR or progressing to T2D. Evidence suggests that lower fasting glucose, greater insulin secretion, younger age, weight loss, and adherence to lifestyle interventions are associated with a higher probability of reverting to NGR (26, 28). Notably, both conventional lifestyle management (CLM) and intensive lifestyle interventions (ILM), incorporating a healthy diet and physical activity, have been demonstrated to restore NGR and lower the risk of progression to T2D in individuals with Pre-DM (29-32). Because of distinct pathophysiology (17, 18), Pre-DM phenotypes exhibit differences in their responsiveness to lifestyle and pharmacological interventions (33, 34). In iIGT, where insulin resistance is predominant, interventions enhancing insulin sensitivity are usually more effective, whereas in iIFG, strategies supporting β-cell function may offer greater benefit (33, 34). Due to the heterogeneous pathophysiology of iIFG and iIGT (16, 17), responses to interventions are likely to be heterogeneous across the Pre-DM spectrum (33, 35-37). Lifestyle interventions are more effective in reducing T2D risk in the iIGT phenotype (35-37), with limited efficacy in individuals with iIFG (34). In the Diabetes Community Lifestyle Improvement Program (DCLIP), the iIFG group did not experience a significant 3-year T2D risk reduction (RRR = 12%, 95% CI = -80% to 57%), whereas participants with combined IGT-IFG showed substantial improvement (RRR = 36%, 95% CI = 3% to 57%) (38). A pooled analysis of four randomized controlled trials indicated that 2.5 years of conventional lifestyle intervention reduced T2D incidence by 3%, 35%, and 49% in the iIFG, iIGT, and IFG-IGT groups, respectively (33).

Table 1 presents the determinants of Pre-DM regression and progression in the TLGS population.

**Table 1.** Determinants of Prediabetes Regression and Progression ^a^

Variables	Regression to NGR	Progression to T2D
**Age (y)**	0.93 (0.90 - 0.97) ^b^	1.02 (1.01 - 1.04) ^c^
**Sex (women)**	1.22 (0.98 - 1.51) ^c^	-
**BMI (kg/m** ^**2**^ **)**	0.97 (0.95 - 1.00) ^b^	1.01 (1.00 - 1.02) ^c^
**HDL-C (mg/dL)**	1.70 (1.23 - 2.34) ^c^	0.73 (0.51 - 1.05) ^b^
**FHD **	0.67 (0.52 - 0.86) ^b^	1.55 (1.27 - 1.90) ^c^
**Education levels (> 12 y)**	2.10 (1.19 - 3.70) ^c^	-
**Smoking**	1.31 (0.99 - 1.74) ^c^	-
**ATD**	0.74 (0.55 - 0.99) ^b^	1.45 (1.08 - 1.93) ^c^
**3-year BW loss**		
< 5% of initial BW	1.32 (1.03 - 1.69) ^c^	-
≥ 5% of initial BW	2.84 (1.97 - 4.11) ^c^	-
**PAL (MET-min/week)**		
600 - 1500	1.32 (0.81 - 2.16) ^d^	1.35 (0.83 - 2.19) ^d^
≥ 1500	1.58 (1.03 - 2.40) ^c^	1.45 (0.96 - 2.20) ^d^
Each 500	1.05 (1.01 - 1.11) ^c^	1.03 (0.98 - 1.08) ^d^

Abbreviations: NGR, normal glucose regulation; T2D, type 2 diabetes; ATD, adipose tissue dysfunction; BMI, Body Mass Index; FHD, family history of diabetes; MET, metabolic equivalent of task.

^a^ Data are relative risk ratio (RRR) or hazard ratio (HR) with 95% confidence interval (CI) derived from the full-adjusted multinomial regression analysis or proportional hazard Cox regression, respectively.

^b^ Decreased probability/risk.

^c^ Increased probability/risk.

^d^ No statistically significant association.

Regression to normoglycemia decreased with increasing age (RRR = 0.97, 95% CI = 0.95 - 0.99), but was more likely in women (RRR = 1.72, 95% CI = 1.18 - 2.50) and in individuals with higher education levels (≥ 12 years; RRR = 2.10, 95% CI = 1.19 - 3.70) (26). Combined IFG-IGT reduced the likelihood of regression compared with isolated IFG (RRR = 0.45, 95% CI = 0.29 - 0.70). The risk of progression to T2D was higher in participants with combined IFG-IGT [hazard ratio (HR) = 1.45, 95% CI = 1.18 - 1.77], elevated Body Mass Index (BMI, HR = 1.07 per kg/m², 95% CI = 1.04 - 1.10), and a family history of diabetes (HR = 1.60, 95% CI = 1.20 - 2.13) (26). Conversely, progression to T2D was associated with higher BMI (RRR = 1.10, 95% CI = 1.05 - 1.15), larger waist circumference (RRR = 0.97, 95% CI = 0.96 - 0.99), and family history of diabetes (RRR = 1.62, 95% CI = 1.07 - 2.45) (26). A 9-year follow-up in the TLGS showed that weight loss of < 5% and ≥ 5% increased the probability of regression to NGR (RRR = 1.44, 95% CI = 1.05 - 1.98 and 2.64, 95% CI = 1.63 - 4.28, respectively), with similar associations observed for reductions in BMI ≥ 5% (RRR = 1.63, 95% CI = 1.01 - 2.64) and waist circumference ≥ 5% (RRR = 1.69, 95% CI = 1.20 - 2.37) (39). Participants with ≥ 5% weight loss had lower mean FSG (111 vs. 116 and 112 mg/dL) and 2h-SG (154 vs. 165 and 168 mg/dL) compared with those who had < 5% weight loss or had weight gain (39). Over three years, participants achieving ≥ 5% weight loss had the highest probability of NGR if they had iIGT, compared to IFG-IGT and iIFG phenotypes (HR = 4.29 vs. 3.90 and 2.84, respectively). A body weight reduction of under 5% modestly increased the probability of Pre-DM regression in the iIGT group (HR = 1.61, 95% CI = 1.03 - 2.52) but did not show benefits for iIFG or IFG-IGT phenotypes (40).

Beyond standard adiposity metrics, adipose tissue dysfunction (ATD), defined according to age-specific Visceral Adiposity Index thresholds, plays a key role in modulating the risk of Pre-DM progression and the likelihood of regression (41). Compared with mild-to-moderate ATD, severe ATD in Pre-DM was associated with a 45% higher risk of T2D onset and a 26% lower likelihood of restoring NGR (RRR = 1.45, 95% CI = 1.08 - 1.93; RRR = 0.74, 95% CI = 0.55 - 0.99) (41). Those with severe ATD also exhibited higher mean FSG (111 vs. 106 mg/dL, 95% CI = 109 - 112 vs. 105 - 108) and 2h-SG concentrations (165 vs. 153 mg/dL, 95% CI = 161 - 168 vs. 149 - 156) over time (41).

Physical activity was also a strong predictor of Pre-DM regression; participants with activity levels > 1,500 metabolic equivalent of task (MET)-min/week had a 58% higher chance of returning to NGR compared with those < 600 MET-min/week (RRR = 1.58, 95% CI = 1.03 - 2.40), and each 500 MET-min/week increment corresponded to a 5% increased probability of Pre-DM regression (RRR = 1.05, 95% CI = 1.01 - 1.11) (42).

In a pragmatic community trial focused on educational interventions targeting lifestyle changes, including 2,073 participants with Pre-DM (761 intervention, 1,312 control), men in the intervention group had a 53% higher chance of returning to NGR after three years (RRR = 1.53, 95% CI = 1.11 - 2.10), with this effect persisting at six years; they also had an increased risk of developing T2D (RRR = 1.53, 95% CI = 1.02 - 2.31) at three years (43). In women, the intervention group had a 1.3-fold higher chance of reversion to NGR at three years (95% CI = 1.00 - 1.69), which disappeared after adjusting for covariates or at six years (43).

To sum up, findings from the TLGS cohort underscore that both non-modifiable factors, including age, sex, and Pre-DM phenotype, as well as modifiable factors, including adiposity measures and physical activity level, interactively determine the Pre-DM transition, highlighting the critical importance of targeted lifestyle interventions to promote regression to NGR and prevent progression to T2D.

### 3.3. Dietary Determinants of Prediabetes Progression and Regression

To the best of our knowledge, the TLGS is the first population-based study to specifically examine the role of dietary factors in Pre-DM transition (Table 2). Various dietary exposures have been examined in relation to Pre-DM outcomes, including overall dietary pattern scores (44), adherence to the Alternate Healthy Eating Index (AHEI) (45), dietary mineral patterns (46), dairy product intake (47, 48), total fat and fatty acid intakes (49), dietary nitrate and nitrite intake (50), and habitual coffee drinking (51). These studies have examined the potential association of dietary factors with regression to NGR or progression to T2D, both in individuals with Pre-DM overall and across specific Pre-DM phenotypes.

**Table 2.** Dietary Determinants of Prediabetes Regression and Progression ^a, b^

Variables	Regression to NGR	Progression to T2D
**Western-style pattern**	1.03 (0.75 - 1.41) ^c^	1.38 (1.00 - 1.89) ^d^
**DASH-style pattern**	0.98 (0.92 - 1.06) ^c^	1.02(0.96 - 1.09) ^c^
**Mediterranean-style pattern**	1.02 (0.86 - 1.29) ^c^	1.06 (0.86 - 1.29) ^c^
**Traditional-healthy pattern**	0.84 (0.63 - 1.13) ^c^	0.80 (0.59 - 1.08) ^c^
**Adherence to AHEI**	2.33 (1.27 - 4.30) in IFG-IGT ^d^	-
**Low-fat diet**	1.44 (1.05 - 1.99) in iIGT ^d^	-
**Mixed-fat pattern**	0.71 (0.50 - 0.99) in iIFG ^e^	-
**ɷ3-fat pattern**	2.29 (1.00 - 5.29) in IFG-IGT ^d^	-
**Total dairy**	1.13 (0.84 - 1.54) ^c^	1.07 (0.79 - 1.44) ^c^
**Low-fat dairy**	0.99 (0.67–1.46) ^c^	0.94 (0.64 - 1.38) ^c^
**High-fat dairy**	1.69 (1.00 - 2.86) ^d^	1.28 (0.75 - 2.17) ^c^
**Milk**	1.34 (0.62 - 2.94) ^c^	0.80 (0.38 - 1.66) ^c^
**Yogurt**	1.82 (1.20 - 2.74) ^d^	1.33 (0.89 - 1.99) ^c^
**Cheese**	1.35 (0.63 - 2.81) ^c^	2.08 (1.01 - 4.30) ^d^
**Cream-butter**	1.10 (0.83 - 1.46) ^c^	0.96 (0.67 - 1.23) ^c^
**Coffee dinking**	2.26 (1.03 - 4.94) ^d^	1.57 (0.71 - 3.45) ^c^
**High-NO3 diet**	-	1.69 (1.04 - 2.74) in iIFG ^d^
**High-NO2 diet**	-	2.07 (1.29 - 3.32) ^d^
**Cr-Se mineral pattern**	1.26 (1.02 - 1.55) ^d^	0.83 (0.62 - 1.11) ^c^
**Fe-Mn mineral pattern**	1.42 (1.14 - 1.76) ^d^	0.67 (0.49 - 0.92) ^e^
**Multi-mineral pattern**	0.98 (0.80 - 1.20) ^c^	0.88 (0.58 - 1.35) ^c^
**High-serum zinc**	-	0.48 (0.23 - 1.01) in iIFG ^e^, 2.44 (1.05 - 5.69) in IFG-IGT ^d^

Abbreviations: NGR, normal glucose regulation; T2D, type 2 diabetes; AHEI, Alternate Healthy Eating Index; IFG-IGT, iIFG-impaired glucose tolerance; iIGT, isolated impaired glucose tolerance; iIFG, isolated impaired fasting glucose; Cr, chromium; Se, selenium; Fe, iron; Mn, manganese.

^a^ Data are relative risk ratio (RRR) or hazard ratio (HR) with 95% confidence interval (CI) derived from the full-adjusted multinomial regression analysis or proportional hazard Cox regression, respectively.

^b^ Mixed fat pattern was identified by a higher load of saturated fat, cholesterol, oleic and linoleic acids, and trans fatty acids. ɷ3 Fat Pattern was primarily characterized by high loadings of docosahexaenoic (DHA), eicosapentaenoic (EPA), and α-linolenic acids (ALA).

^c^ No statistically significant association.

^d^ Increased probability/risk.

^e^ Decreased probability/risk.

In the TLGS cohort of adults with Pre-DM, adherence to a Western-style dietary pattern (i.e., defined by greater intake of red meats, hydrogenated fats, sodium, and total fat intakes) was associated with a higher risk of progression to T2D (RRR = 1.38, 95% CI = 1.00 - 1.89, P = 0.050), while the healthy and processed-foods dietary patterns were not significantly associated with progression to T2D or regression to NGR (44). High adherence to the AHEI was associated with an increased probability of regression to normal glucose regulation (HR = 1.31, 95% CI = 1.13 - 1.51) (45). Among Pre-DM phenotypes, individuals with combined IFG-IGT exhibited the greatest benefit from high AHEI adherence compared with those with isolated phenotypes (HR = 2.33, 95% CI = 1.27 - 4.30). Additionally, the mean estimated time to reversion to NGR was shorter among individuals with IFG-IGT who demonstrated high adherence to the AHEI compared with those with low adherence (6.2 vs. 8.4 years, Plog-rank = 0.051) (45).

Adherence to a low-fat diet (LFD) was associated with an increased likelihood of Pre-DM reversion in individuals with the iIGT phenotype (HR = 1.44, 95% CI = 1.05 - 1.98) (49). A higher score in the mixed fat pattern (MFP, characterized by higher load of saturated fat, cholesterol, oleic, linoleic, and trans fatty acids) was inversely associated with reversion to NGR in both isolated iIFG and iIGT phenotypes (HR = 0.71, 95% CI = 0.50 - 0.99, and HR = 0.60, 95% CI = 0.37 - 0.97, respectively) (49). Higher adherence to the ω3 Fat Pattern [ω3FP, rich in docosahexaenoic (DHA), eicosapentaenoic (EPA), and α-linolenic acids (ALA)] was associated with a twofold higher incidence of NGR in individuals with combined IFG-IGT (HR = 2.29, 95% CI = 1.00 - 5.29) (49).

We further showed that higher intake of high-fat dairy was associated with increased probability of returning to NGR (RRR = 1.69, 95% CI = 1.00-2.86, P = 0.05, per 200 g/day) (47). Specifically, higher yogurt consumption was associated with a higher chance of Pre-DM regression (RRR = 1.82, 95% CI = 1.20-2.74, P = 0.01) (47). In terms of changes in dairy consumption, a reduction in total dairy intake exceeding 0.5 serving/day was associated with a significantly higher risk of progression to T2D [odds ratio (OR) = 1.56, 95% CI = 1.02 - 2.41], whereas an increase in low-fat dairy intake by 0.5 serving/day corresponded to a significantly lower T2D incidence in individuals with Pre-DM (OR = 0.56, 95% CI = 0.35 - 0.90) (48). Specifically, higher intake of low-fat milk (OR = 0.59, 95% CI = 0.37 - 0.92) and low-fat yogurt (OR = 0.55, 95% CI = 0.33 - 0.93) was protective, whereas replacing these with regular cheese increased T2D risk by 66% and 47%, respectively (48).

We also showed that coffee consumption was associated with a higher likelihood of returning to NGR (RRR = 2.26, 95% CI = 1.03 - 4.97), and coffee drinkers exhibited significantly lower 2h-SG concentrations over time compared with non-drinkers (152 mg/dL, 95% CI = 144 - 159 vs. 162 mg/dL, 95% CI = 155 - 169, P = 0.05) (51).

Excessive dietary exposure to nitrate (≥ 645 mg/d) and nitrite (≥ 11.5 mg/d) has also been associated with an increased risk of developing T2D specifically among those with the iIFG phenotype (50). Every 100 mg/d of nitrate and 2 mg/d nitrite intake exceeding the allowance daily intake (ADI, i.e., 3.7 and 0.06 mg/kg body weight ~260 and 4.2 mg for an adult weighing 70 kg) was associated with an increased risk of progression from iIFG to T2D by 13% and 25%, respectively (50).

Mineral status, assessed through food frequency questionnaires or serum biomarkers, was also associated with Pre-DM transition. A principal factor analysis on dietary mineral intakes showed that higher adherence to chromium-selenium (Cr-Se) and iron-manganese (Fe-Mn) mineral patterns was associated with greater likelihood of regression to NGR, with HR of 1.26 (95% CI = 1.02 - 1.55) and 1.42 (95% CI = 1.14 - 1.76), respectively. In addition, the Fe-Mn pattern was associated with a reduced risk of developing T2D (HR = 0.67, 95% CI = 0.49 - 0.92) (46). In individuals with iIGT, serum zinc concentrations were positively associated with incidence of T2D (HR = 2.27, 95% CI = 1.01 - 5.10, and HR = 2.44, 95% CI = 1.05 - 5.69 for serum zinc ≥ 106 and 134 µg/dL, respectively) (52). Serum zinc concentrations ≥ 122 μg/dL appeared to reduce T2D risk by 52% in men with iIFG (HR = 0.48, 95% CI = 0.23 - 1.01, P = 0.052), but no effect was noted in women (53).

Taken together, findings from the TLGS suggest that dietary patterns and mineral status exert measurable effects on Pre-DM transitions, either promoting regression to normoglycemia or accelerating progression to T2D, with effects that appear to vary across specific Pre-DM phenotypes.

## 4. Conclusions

**Figure 1.**
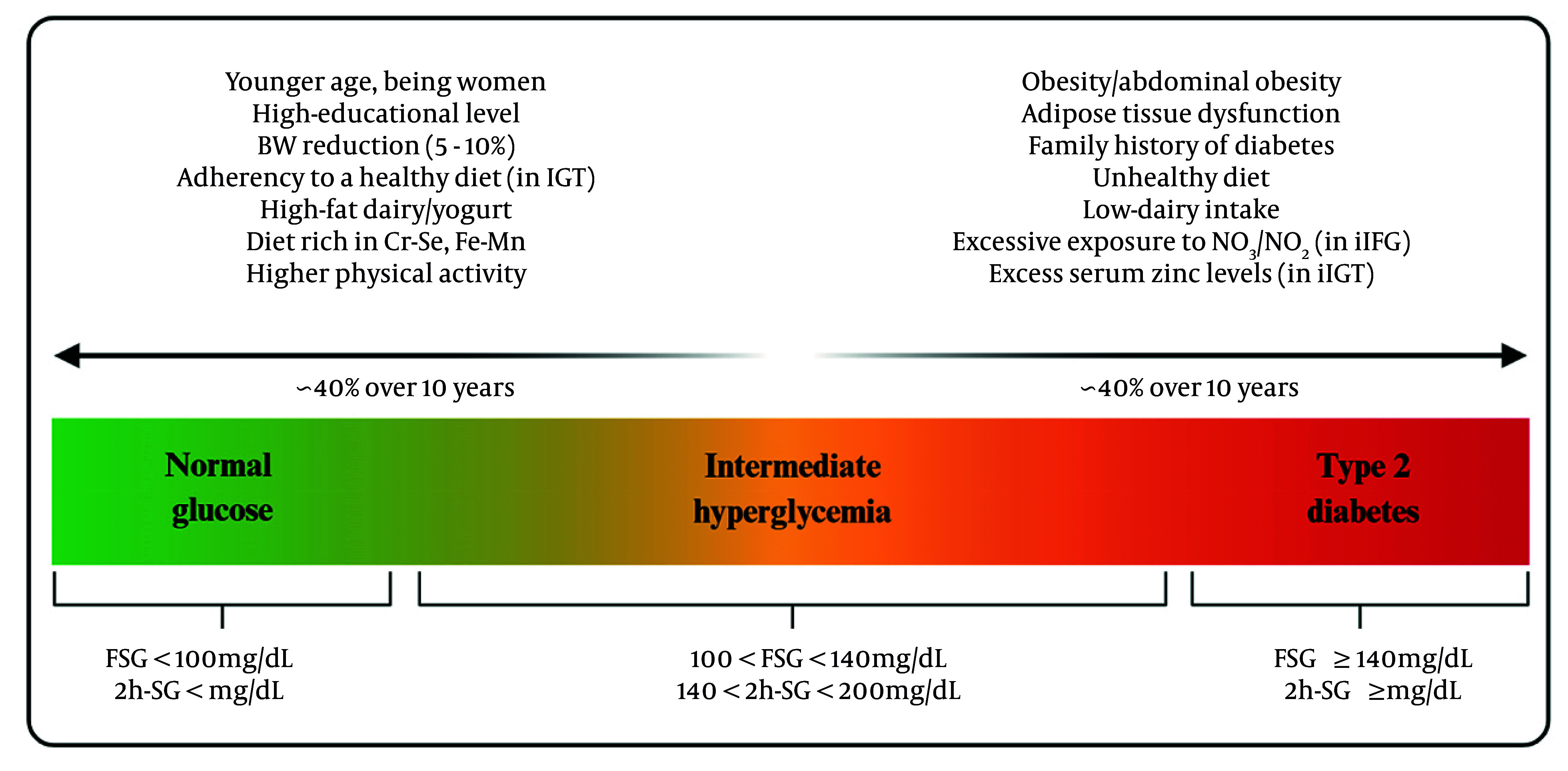
Modifiable and non-modifiable determinants of prediabetes (Pre-DM) transition identified in the Tehran Lipid and Glucose Study (TLGS) population (abbreviations: FSG, fasting serum glucose; 2h-SG, 2-hour serum glucose; iIFG, isolated impaired fasting glucose; iIGT, isolated impaired glucose tolerance; Cr, chromium; Se, selenium; Fe, iron; Mn, manganese).

The findings from the TLGS shed light on the complex factors influencing transitions in Pre-DM. Both non-modifiable factors, including age, family history, and glycemic phenotype, and modifiable factors, including body weight, adiposity distribution, physical activity, and dietary patterns, affect the Pre-DM transition. Figure 1 illustrates modifiable and non-modifiable determinants of Pre-DM regression and progression, identified in the TLGS population. Analysis by specific phenotypes shows that subjects with iIGT and combined IFG-IGT are more likely to return to NGR in response to weight loss and healthy eating. The isolated iIFG phenotype appears particularly susceptible to excessive exposure to nitrate/nitrite, zinc, and diets high in cholesterol, saturated, and trans fats, which may attenuate reversion to NGR and facilitate progression to T2D. These insights emphasize the importance of developing targeted strategies based on these phenotypes to help prevent the progression to T2D and encourage a return to NGR. Lifestyle changes, including moderate weight loss, increased exercise, and adherence to better dietary patterns — especially those rich in essential minerals — can make a real difference for those who are more responsive to these changes.

Public health initiatives should consider these findings and provide guidance tailored to different phenotypes to enhance the effectiveness of prevention programs for individuals at high risk of T2D. More research is definitely needed to better understand why different phenotypes react differently to interventions, particularly regarding insulin resistance and β-cell function. Long-term studies examining personalized lifestyle and dietary interventions, particularly for individuals with isolated iIFG, could provide valuable insights. Additionally, exploring the interaction between genetics, environment, and diet could help refine our understanding and lead to more effective prevention strategies for managing and reversing Pre-DM.

## Data Availability

The dataset presented in the study is available on request from the corresponding author during submission or after publication.

## References

[1] American Diabetes Association (2021). Classification and Diagnosis of Diabetes: Standards of Medical Care in Diabetes-2021.. Diabetes Care..

[2] Faerch K, Borch-Johnsen K, Holst JJ, Vaag A (2009). Pathophysiology and aetiology of impaired fasting glycaemia and impaired glucose tolerance: does it matter for prevention and treatment of type 2 diabetes?. Diabetologia..

[3] Hostalek U (2019). Global epidemiology of prediabetes - present and future perspectives.. Clin Diabetes Endocrinol..

[4] Hadaegh F, Derakhshan A, Zafari N, Khalili D, Mirbolouk M, Saadat N (2017). Pre-diabetes tsunami: incidence rates and risk factors of pre-diabetes and its different phenotypes over 9 years of follow-up.. Diabet Med..

[5] Sallar A, Dagogo-Jack S (2020). Regression from prediabetes to normal glucose regulation: State of the science.. Exp Biol Med..

[6] Tabak AG, Herder C, Rathmann W, Brunner EJ, Kivimaki M (2012). Prediabetes: a high-risk state for diabetes development.. Lancet..

[7] Rooney MR, Fang M, Ogurtsova K, Ozkan B, Echouffo-Tcheugui JB, Boyko EJ (2023). Global Prevalence of Prediabetes.. Diabetes Care..

[8] Mahtab N, Farzad H, Mohsen B, Nakisa D (2017). The 10-year trend of adult diabetes, prediabetes and associated risk factors in Tehran: Phases 1 and 4 of Tehran Lipid and Glucose Study.. Diabetes Metab Syndr..

[9] Nikkhah A, Farzi Y, Azizpour Y, Rezaei N, Mirzad M, Shahrestanaki SK (2025). Prevalence, awareness, treatment, and control of prediabetes and diabetes mellitus in older adults: findings from Iranian STEPS surveys (2016 and 2021).. BMC Public Health..

[10] The Lancet Diabetes E (2025). Prediabetes: much more than just a risk factor.. Lancet Diabetes Endocrinol..

[11] Moslehi N, Mahdavi M, Mirmiran P, Azizi F (2024). Ultra-processed foods and the incidence of pre-diabetes and type 2 diabetes among Iranian adults: the Tehran lipid and glucose study.. Nutr Metab (Lond)..

[12] Noroozzadeh M, Mousavi M, Naz MSG, Farahmand M, Azizi F, Ramezani Tehrani F (2025). Early menopause in mothers and the risks of pre-diabetes and type 2 diabetes mellitus in female and male offspring: a population-based cohort study.. Reprod Biol Endocrinol..

[13] Rahmati M, Saei Ghare Naz M, Azizi F, Ramezani Tehrani F (2022). Pregnancy loss and subsequent risk of prediabetes, diabetes and metabolic syndrome in couples: Tehran lipid and glucose study.. J Transl Med..

[14] Azizi F, Takyar M, Zadeh-Vakili A (2018). Contributions and Implications of the Tehran Lipid and Glucose Study.. Int J Endocrinol Met..

[15] Davidson MB (2022). Historical review of the diagnosis of prediabetes/intermediate hyperglycemia: Case for the international criteria.. Diabetes Res Clin Pract..

[16] Kanat M, Mari A, Norton L, Winnier D, DeFronzo RA, Jenkinson C (2012). Distinct beta-cell defects in impaired fasting glucose and impaired glucose tolerance.. Diabetes..

[17] Abdul-Ghani MA, Tripathy D, DeFronzo RA (2006). Contributions of beta-cell dysfunction and insulin resistance to the pathogenesis of impaired glucose tolerance and impaired fasting glucose.. Diabetes Care..

[18] Richter B, Hemmingsen B, Metzendorf MI, Takwoingi Y (2018). Development of type 2 diabetes mellitus in people with intermediate hyperglycaemia.. Cochrane Database Syst Rev..

[19] Gerstein HC, Santaguida P, Raina P, Morrison KM, Balion C, Hunt D (2007). Annual incidence and relative risk of diabetes in people with various categories of dysglycemia: a systematic overview and meta-analysis of prospective studies.. Diabetes Res Clin Pract..

[20] Derakhshan A, Tohidi M, Arshi B, Khalili D, Azizi F, Hadaegh F (2015). Relationship of hyperinsulinaemia, insulin resistance and beta-cell dysfunction with incident diabetes and pre-diabetes: the Tehran Lipid and Glucose Study.. Diabet Med..

[21] Hanefeld M, Koehler C, Fuecker K, Henkel E, Schaper F, Temelkova-Kurktschiev T (2003). Insulin secretion and insulin sensitivity pattern is different in isolated impaired glucose tolerance and impaired fasting glucose: the risk factor in Impaired Glucose Tolerance for Atherosclerosis and Diabetes study.. Diabetes Care..

[22] Nathan DM, Davidson MB, DeFronzo RA, Heine RJ, Henry RR, Pratley R (2007). Impaired fasting glucose and impaired glucose tolerance: implications for care.. Diabetes Care..

[23] Varghese RT, Dalla Man C, Sharma A, Viegas I, Barosa C, Marques C (2016). Mechanisms Underlying the Pathogenesis of Isolated Impaired Glucose Tolerance in Humans.. J Clin Endocrinol Metab..

[24] Masrouri S, Tamehri Zadeh SS, Tohidi M, Azizi F, Hadaegh F (2024). Linking extent of return to fasting state after oral glucose tolerance test to future risk of prediabetes and type 2 diabetes: Insights from the TLGS.. J Diabetes Investig..

[25] Kohansal K, Ahmadi N, Hadaegh F, Alizadeh Z, Azizi F, Habibi-Moeini AS (2022). Determinants of the progression to type 2 diabetes and regression to normoglycemia in people with pre-diabetes: A population-based cohort study over ten years.. Prim Care Diabetes..

[26] Alizadeh Z, Baradaran HR, Kohansal K, Hadaegh F, Azizi F, Khalili D (2022). Are the determinants of the progression to type 2 diabetes and regression to normoglycemia in the populations with pre-diabetes the same?. Front Endocrinol (Lausanne)..

[27] van Herpt TTW, Ligthart S, Leening MJG, van Hoek M, Lieverse AG, Ikram MA (2020). Lifetime risk to progress from pre-diabetes to type 2 diabetes among women and men: comparison between American Diabetes Association and World Health Organization diagnostic criteria.. BMJ Open Diabetes Res Care..

[28] Perreault L, Kahn SE, Christophi CA, Knowler WC, Hamman RF, Diabetes Prevention Program Research G (2009). Regression from pre-diabetes to normal glucose regulation in the diabetes prevention program.. Diabetes Care..

[29] Perreault L, Pan Q, Mather KJ, Watson KE, Hamman RF, Kahn SE (2012). Effect of regression from prediabetes to normal glucose regulation on long-term reduction in diabetes risk: results from the Diabetes Prevention Program Outcomes Study.. The Lancet..

[30] Rasaei N, Fallah M, Gholami F, Karimi M, Noori S, Bahrampour N (2023). The association between glycemic index and glycemic load and quality of life among overweight and obese women: a cross-sectional study.. BMC Nutr..

[31] Shiva F, Nourimajd S, Asadi S, Rasaei N, Hasanzadeh M, Qorbani M (2022). Association of dietary acid-base load and diabetic sensorimotor polyneuropathy in patients with type 2 diabetes mellitus: A case-control study.. Clin Nutr ESPEN..

[32] Zamani M, Nikbaf-Shandiz M, Aali Y, Rasaei N, Zarei M, Shiraseb F (2023). The effects of acarbose treatment on cardiovascular risk factors in impaired glucose tolerance and diabetic patients: a systematic review and dose-response meta-analysis of randomized clinical trials.. Front Nutr..

[33] Sathish T, Khunti K, Narayan KMV, Mohan V, Davies MJ, Yates T (2023). Effect of Conventional Lifestyle Interventions on Type 2 Diabetes Incidence by Glucose-Defined Prediabetes Phenotype: An Individual Participant Data Meta-analysis of Randomized Controlled Trials.. Diabetes Care..

[34] Campbell MD, Sathish T, Zimmet PZ, Thankappan KR, Oldenburg B, Owens DR (2020). Benefit of lifestyle-based T2DM prevention is influenced by prediabetes phenotype.. Nat Rev Endocrinol..

[35] Lindström J, Ilanne-Parikka P, Peltonen M, Aunola S, Eriksson JG, Hemiö K (2006). Sustained reduction in the incidence of type 2 diabetes by lifestyle intervention: follow-up of the Finnish Diabetes Prevention Study.. The Lancet..

[36] Pan XR, Li GW, Hu YH, Wang JX, Yang WY, An ZX (1997). Effects of diet and exercise in preventing NIDDM in people with impaired glucose tolerance. The Da Qing IGT and Diabetes Study.. Diabetes Care..

[37] Li G, Zhang P, Wang J, Gregg EW, Yang W, Gong Q (2008). The long-term effect of lifestyle interventions to prevent diabetes in the China Da Qing Diabetes Prevention Study: a 20-year follow-up study.. The Lancet..

[38] Weber MB, Ranjani H, Staimez LR, Anjana RM, Ali MK, Narayan KM (2016). The Stepwise Approach to Diabetes Prevention: Results From the D-CLIP Randomized Controlled Trial.. Diabetes Care..

[39] Masumi M, Bahadoran Z, Mirmiran P, Khalili D, Sarvghadi F, Azizi F (2024). Effect of 3-year changes in adiposity measures on the pre-diabetes regression and progression: a community-based cohort study.. BMC Public Health..

[40] Bahadoran Z, Mirmiran P, Azizi F, Hosseinpanah F (2024). The association of body weight change and regression to normoglycemia in different phenotypes of pre-diabetes: Findings of a longitudinal cohort study.. Clin Nutr ESPEN..

[41] Jalali M, Bahadoran Z, Mirmiran P, Azizi F, Hosseinpanah F (2024). Severity of adipose tissue dysfunction is associated with progression of pre-diabetes to type 2 diabetes: the Tehran Lipid and Glucose Study.. BMC Public Health..

[42] Bahadoran Z, Mirmiran P, Shabani M, Azizi F (2023). Higher daily physical activity levels may facilitate pre-diabetes regression to normoglycemia: A longitudinal study among an Iranian population.. Prev Med Rep..

[43] Khalili D, Dehghani Z, Asgari S, Hadaegh F, Azizi F (2024). Progression to diabetes and regression to normoglycemia in pre-diabetic subjects: results from a pragmatic community trial in a middle-income country.. J Diabetes Metab Disord..

[44] Mirmiran P, Hosseini S, Bahadoran Z, Azizi F (2023). Dietary pattern scores in relation to pre-diabetes regression to normal glycemia or progression to type 2 diabetes: a 9-year follow-up.. BMC Endocr Disord..

[45] Bahadoran Z, Mirmiran P, Azizi F (2025). Is adherence to the alternate healthy eating index an effective approach for prediabetes reversion? A longitudinal follow-up.. Clin Nutr ESPEN..

[46] Jalali M, Bahadoran Z, Mirmiran P, Azizi F (2025). Dietary mineral patterns are associated with the pre-diabetes regression and progression: the Tehran lipid and glucose study (TLGS).. BMC Nutr..

[47] Bahadoran Z, Mirmiran P, Azizi F (2024). Usual intake of dairy products and the chance of pre-diabetes regression to normal glycemia or progression to type 2 diabetes: a 9-year follow-up.. Nutr Diabetes..

[48] Yuzbashian E, Asghari G, Mirmiran P, Chan CB, Azizi F (2021). Changes in dairy product consumption and subsequent type 2 diabetes among individuals with prediabetes: Tehran Lipid and Glucose Study.. Nutr J..

[49] Bahadoran Z, Kashani Z, Mahdavi M, Mirmiran P, Azizi F (2025). Total fat intake and fatty acid patterns and prediabetes regression: differential effects across phenotypes in a population-based cohort.. Eur J Med Res..

[50] Bahadoran Z, Mirmiran P, Ghasemi A, Azizi F (2025). Excessive exposure to nitrate and nitrite boosts progression of isolated impaired fasting glucose to type 2 diabetes: a cohort study.. J Diabetes Metab Disord..

[51] Hosseini S, Bahadoran Z, Mirmiran P, Azizi F (2024). Habitual coffee drinking and the chance of prediabetes remission: findings from a population with low coffee consumption.. J Diabetes Metab Disord..

[52] Bahadoran Z, Ghafouri-Taleghani F, Azizi F, Ghasemi A (2025). High Serum Zinc Concentration Accelerates Progression of Isolated Impaired Glucose Tolerance to Type 2 Diabetes: A Cohort Study.. Biol Trace Elem Res..

[53] Bahadoran Z, Azizi F, Ghasemi A (2025). Sex-Specific Association Between Serum Zinc Concentration and Risk of Developing Type 2 Diabetes in Individuals with Isolated Impaired Fasting Glucose: A Prospective Cohort.. Biol Trace Elem Res..

